# Cuproptosis related gene PDHB is identified as a biomarker inversely associated with the progression of clear cell renal cell carcinoma

**DOI:** 10.1186/s12885-023-11324-0

**Published:** 2023-08-28

**Authors:** Hu Wang, Zhan Yang, Xingyu He, Fengran Guo, Hao Sun, Sen Xu, Chao Xu, Zhu Wang, Hongzhuang Wen, Zhihai Teng, Yaxuan Wang, Zhenwei Han

**Affiliations:** Department of Urology, The Second Hospital of Hebei Medical University, 215 West Heping Road, Shijiazhuang, 050000 China

**Keywords:** Cuproptosis, PDHB, Clear cell renal cell carcinoma, Tumor immune microenvironment

## Abstract

**Background:**

Cuproptosis is a newly discovered programmed cell death dependent on mitochondrial respiratory disorder induced by copper overload. Pyruvate dehydrogenase E1 subunit beta (PDHB) is one of the cuproptosis genesand is a nuclear-encoded pyruvate dehydrogenase, which catalyzes the conversion of pyruvate to acetyl coenzyme A. However, the mechanism of PDHB in clear cell renal cell carcinoma (ccRCC) remains unclear.

**Methods:**

We used data from TCGA and GEO to assess the expression of PDHB in normal and tumor tissues. We further analyzed the relationship between PDHB and somatic mutations and immune infiltration. Finally, we preliminarily explored the impact of PDHB on ccRCC.

**Results:**

The expression level of PDHB was lower in tumor tissue compared with normal tissue. Meanwhile, the expression level of PDHB was also lower in high-grade tumors than low-grade tumors. PDHB is positively correlated with prognosis in ccRCC. Furthermore, PDHB may be associated with decreased risk of VHL, PBRM1 and KDM5C mutations. In 786-O cells, copper chloride could promote the expression of cuproptosis genes (DLAT, PDHB and FDX1) and inhibit cell growth. Last but not least, we found that PDHB could inhibit the proliferation and migration of ccRCC cells.

**Conclusion:**

Our results demonstrated that PDHB could inhibit the proliferation, migration and invasion in ccRCC cells, which might be a prognostic predictor of ccRCC. Targeting this molecular might provide a new therapeutic strategy for patients with advanced ccRCC.

**Supplementary Information:**

The online version contains supplementary material available at 10.1186/s12885-023-11324-0.

## Introduction

Renal cell carcinoma (RCC) is a kind of malignant tumor originating from the tubular epithelial system of the kidney, ranking the second place in the incidence of urological malignancies [1]. The clear cell renal cell carcinoma (ccRCC) is the most common renal cell carcinoma, accounting for 70–80% of the cases. Its etiology is complex and varied, which might be related to genetics, smoking, obesity, hypertension and antihypertensive therapy [2, 3]. The early symptoms of ccRCC are not typical, many patients were diagnosed at an advanced stage [4]. ccRCC is resistance to radiotherapy and chemotherapy, and tyrosine kinase inhibitors (TKIs), immunotherapy and immune combination targeted therapy are new trends in advanced ccRCC treatment [5],. However, there are still many disadvantages, such as high costs, drug resistance, and off-target effects. Therefore, it is an urgent need to find new markers to guide the treatment of ccRCC.

Copper holds a pivotal role as a crucial metallic element within living organisms [6], intricately intertwined with a multitude of biological functions. These functions encompass an array of vital processes such as mitochondrial respiration, iron absorption, antioxidant defense, and detoxification mechanisms [7]. So far, dysregulation of copper ion homeostasis has been reported to be associated with Alzheimer’s disease, blood diseases, cardiovascular diseases and cancer [7, 8]. Recent studies have identified a non-apoptotic programmed death (cuproptosis) [9, 10] caused by intracellular copper accumulation, which is different from apoptosis [11], ferroptosis [12], pyrodeath [13] and cell necrosis [14]. The core mechanism underlying cuproptosis hinges on the accumulation of intracellular copper ions. These ions directly engage with lipid acylated constituents of the tricarboxylic acid cycle (TCA), thereby prompting the aggregation and disruption of these crucial proteins. This disruption impedes the smooth functioning of the TCA cycle, culminating in the initiation of proteotoxic stress and ultimately resulting in the activation of cell death pathways [15, 16].

Recent studies have found that cuproptosis is involved in the development of a variety of tumors [17], such as breast cancer [18], kidney cancer [19, 20], bladder cancer [21] and hepatocellular carcinoma [22]. Pyruvate dehydrogenase E1 subunit beta (PDHB) is a cuproptosis gene located in mitochondria, which functions as catalyzing the conversion of glucose-derived pyruvate to acetyl coenzyme A. This process holds significant importance within both glycolysis and tricarboxylic acid cycle [23]. *Zhu* et al. have found that PDHB is involved in regulating cell growth, invasion and metabolism in colon cancer [24]. However, the potential impact of the cuproptosis gene *PDHB* in the progression of renal cell carcinoma and its molecular mechanisms are largely unknown.

In this study, we analyzed The Cancer Genome Atlas (TCGA) public database and Gene Expression Omnibus (GEO) database to validate the expression and clinical significance of PDHB in ccRCC. The prognostic value of PDHB as a cuproptosis gene for ccRCC was uncovered.

## Materials and methods

### Data retrieval and preprocessing

The RNA sequencing expression profiles and survival information for patients with ccRCC in TCGA (https://tcga-data.nci.nih.gov/tcga/, accessed on 12 July 2022) database were obtained from UCSC Xena data portal (https://xenabrowser.net/, accessed on 15 July 2022). 535 tumor samples and 72 normal kidney tissue samples were included from TCGA database. GSE76351 and GSE68417 [25] gene expression matrix were downloaded from GEO (https://www.ncbi.nlm.nih.gov/geo/, accessed on 1 July 2022) database and 20 tumor samples and 47 paracancer samples were included. The microarray platform for GSE76351 and GSE6841 were GPL11532 and GPL6244 respectively. The raw from GEO database was subjected to Log2 transformation.

### Relationship between PDHB and prognosis of ccRCC

We used ggplot2 (v3.3.2) and R software version 4.0.3 to analyze clinical data from TCGA. The *p*-values less than 0.05 were considered to be statistically significant. We evaluated the clinical predictive value of PDHB in multiple cancers using the “survival” package. Receiver Operating characteristic (ROC) curves were used to assess differences of PDHB expression between ccRCC samples and paracancer samples. The higher the value of area under ROC curve (AUC), the higher the corresponding predictive power.

### Construction of PDHB co-expression network and annotation of its associated genes

LinkedOmics (http://www.linkedomics.org) is a public portal containing multi-omics data from all 32 TCGA cancer types and the 10 Clinical Proteomics Tumor Analysis Consortium (CPTAC) [26]. In the “LinkFinder” module of LinkedOmics, we used the Pearson test for statistical analysis of PDHB co-expression, displayed as volcano, heat map or scatter plot. “LinkInterpreter” module of LinkedOmics was used to conduct analyses of Gene Ontology (Biological Process), Kyoto Encyclopedia of Genes and Genomes (KEGG) pathways [27], kinase-target enrichment, miRNA-target enrichment and transcription factor-target enrichment through Gene Set Enrichment Analysis (GSEA). The rank criterion is false discovery rate (FDR) < 0.05.

### Correlation of PDHB with molecular and immunological properties

From the TCGA dataset, we were able to extract the relevant somatic alteration data for TCGA-KIRC cohort. The number of non-synonymous point mutations in somatic cells was then calculated for each sample using the ‘maftools’ package in R software. Tumor Immune Estimation Resource (TIMER, https://cistrome.shinyapps.io/timer/) is a public database for the systematic analysis of different cancer types of immune infiltrates in a comprehensive resource [28]. We used the “GENE” module to explore the correlation between six immune cell infiltrations of B cells, CD4 + T cells, CD8 + T cells, neutrophils, macrophages and dendritic cells in KIRC by tumor purity-corrected partial Spearman’s correlation.

### Establishment and validation of nomogram scoring system

Based on the results of the independent prognostic analysis, the “nomoR“ [29] software package was used to develop nomogram graphs predicting clinical characteristics and risk scores. The characteristics of the patients included T-stage, N-stage, M-stage, pathologic stage and histologic grade. The “timeroc“ [30, 31] software package was used to perform ROC curve scores for 1-year, 3-year and 5-year survival rates. The calibration plots of nomograms are used to depict the predicted values between the predicted 1-year, 3-year and 5-year survival events and the actual observed outcomes.

### Human ccRCC specimens

This study recruited four patients with renal cell carcinoma undergone radical nephrectomy in the Second Hospital of Hebei Medical University. All patients did not receive any treatment prior to surgery. Postoperative pathology confirmed that all cases were ccRCC. Immediately after resection, two different types of tissues from each ccRCC patient were processed, including non-tumor tissue (N) located 2 cm away from the tumor margin and tissue from the tumor (T). Two methods were used to handle each patient’s specimens. One method involved immediate freezing of the excised tissue, which was preserved in liquid nitrogen for further experiments. The other method involved fixation in a 10% formaldehyde solution, followed by embedding in paraffin for subsequent experiments.

### In vitro cell culture/maintenance

The cell lines 769P, 786-O, OSRC2, ACHN, and Caki-1 (obtained from American Type Culture Collection) were cultured using standard methods. The recommended cell culture media were used, supplemented with 10% fetal bovine serum (FBS). The cells were incubated in chambers with an atmosphere of 5% CO2 and 95% air at a temperature of 37 °C.

### The PDHB overexpression plasmid construction

To increase PDHB expression, oe-PDHB (from GenePharma) was used, while a non-specific pcDNA3.1 (from GenePharma) was used as a control. The transfection was performed using Lipofectamine 2000 following the manufacturer’s instructions. The 786-O cell line was incubated with the plasmid complexes for 48 h before proceeding to the next step.

### Detection of mRNA expression by quantitative fluorescence polymerase chain reaction(qRT-PCR)

RNAs were isolated using the Trizol reagent (Invitrogen, Grand Island, NY, USA). RNA was utilized for reverse transcription using Superscript III transcriptase (Invitrogen). In order to measure the mRNA expression level of the target genes, quantitative real-time PCR (qRT-PCR) was carried out using a Bio-Rad CFX96 system with SYBR green. Expression levels were adjusted to account for the expression of GAPDH. The qRT-PCR procedure was as follows: 50 ◦C for two minutes, 95 ◦C for eight minutes and thirty seconds, 45 cycles at 95 ◦C for fifteen seconds each, and 60◦C for one minute; 95◦C for 1 min, 55◦C for 1 min, and 55◦C for 10 s made up the extension. The normalized control was GAPDH. The primer sequences involved are as follows:

PDHB

F: AAGAGGCGCTTTCACTGGAC

R: ACTAACCTTGTATGCCCCATCA

FDX1:

F: TTCAACCTGTCACCTCATCTTTG

R: TGCCAGATCGAGCATGTCATT

DLAT:

F: CGGAACTCCACGAGTGACC

R: CCCCGCCATACCCTGTAGT

ART:

F: GGCCAAAGGCAGTTGTATTGA

R: GTGAGTACCCCAAAAATAGCAGG

FOXMI:

F: CGTCGGCCACTGATTCTCAAA

R: GGCAGGGGATCTCTTAGGTTC

GAPDH

F: GGAGCGAGATCCCTCCAAAAT

R: GGCTGTTGTCATACTTCTCATGG

### Western blotting assay

RIPA whole-cell lysis solution was used to lyse the cells. Total proteins were then extracted, measured, and semi-dry transferred to PVDF (Millipore, Billerica, MA, USA) membranes after separation by 8–12% SDS-PAGE electrophoresis; PVDF membranes were first blocked with TBS + Tween (TBST) solution containing 5% skim milk for 2 h. Then they were washed, and incubated with primary antibody at 4 ◦C overnight (PDHB, 12649-1-AP, Proteintech, 1:50); they were then rewashed and incubated for 2 h with a secondary antibody that was colored by horseradish peroxidase. The PVDF membrane was washed before the chemiluminescent substrate was applied and then developed by a gel imaging system, after which the grayscale values were measured. The membranes were trimmed reasonably before chemiluminescence (the original length is preserved).

### Transwell migration experiments

Cells were collected in serum-free medium and added at 1 × 10^5^/mL to the upper chamber of an 8.0 μm pore size polycarbonate membrane filter (Corning Incorporated, Corning, New York, USA), 600 µL of 10% fetal bovine serum medium was added to the lower chamber and incubated at 37◦C for 36 h. Three replicate wells were set up for each group of experiments, and experiments were repeated three times. The fields of view were randomly selected for counting and photographed at the same time. The number of migrating cells was measured using Image J software.

### Wound-healing assay to detect cell migration

Tumor cells were then added at a density of 1 × 10^5^ cells per well and transfected with oe-PDHB and oe-NC respectively. After the cells had fully adhered to the walls, on the back of a 6-well plate marked with 3 parallel lines. A 10 uL sterile pipette was used to perform the wound-healing Assay. The cells were then washed 3 times with PBS, added to serum-free medium and placed in an incubator at 37 °C. Finally, images were taken at 0 and 24 h. Then the results were analyzed using Image J software.

### Immunohistochemical staining and evaluation

Immunohistochemical staining was performed on the cross-section of 4 μm paraffin-embedded tissues. Sections were removed with xylene, rehydrated, pre-incubated with 10% normal goat serum (710,027, KPL, USA), then incubated overnight with primary antibody (PDHB, 14744-1-AP, protein, 1:200) at 4◦C and washed. This was followed by a secondary antibody (Horseradish peroxide-labeled Rabbit IgG antibody, 021516, KPL, USA). Finally, the sections were re-stained with hematoxylin, then dehydrated, washed and evaluated.

### Cell counting kit-8 (CCK-8) assay

Cells were inoculated into 96-well plates at a rate of 5000 cells/well overnight. Copper chloride solution and elesclomol [32] were added 1:1 to the 96-well plates at various concentrations of medium. Firstly, we used a larger concentration gradient of copper chloride (0,2.5, 5, 10, 20, 40, 80, 160, 320, 640, (nM)), then, we narrowed the concentration gradient (0, 20, 40, 60, 80, 100, (nM)). After 48 h, CCK-8 (Sevenbio, Beijing, China) reagent was added to each well and then incubated at 37 °C for 2 h. The absorbance at 450 nm was read using a Bio-Tek MQX200 micro-plate reader (Bio-Tek Instruments Inc., Winooski, VT, USA). The half maximum inhibitory concentration (half inhibitory concentration) was then calculated.

### Flow cytometry

To conduct cell cycle and apoptosis analysis, 786-O cells were seeded at a density of 3 × 10^5^ cells per well in a 6-well plate following the manufacturer’s protocol. The indicated constructs were transfected or copper chloride was added. After 48 h of transfection, cells were centrifuged at 1000 g for 5 min and fixed overnight in 70% ethanol at 4 °C. Subsequently, a DNA content quantitation assay (cat. no. CA1510-20T; Beijing Solarbio Science & Technology Co., Ltd.) was used to quantify the DNA content. Apoptosis rate of 786-O cells was analyzed using the Annexin V-FITC/PI Apoptosis Detection Kit (cat. no. CA1020-20T; Beijing Solarbio Science & Technology Co., Ltd.) according to the manufacturer’s instructions. Briefly, 5 × 10^5^ cells were centrifuged at 1000 g for 5 min, resuspended in 200 µl of binding buffer, and incubated with 5 µl of Annexin V-FITC and 5 µl of propidium iodide (PI) in the dark at 37 °C for 15 min. Annexin V + PI- cells were considered as early apoptotic cells, while Annexin V + PI + cells were considered as late apoptotic cells. Data acquisition was performed using the FACSVerse flow cytometer (BD Biosciences), and analysis was conducted using FlowJo software (version 10.0.7; FlowJo LLC). Each experiment was performed in triplicate, and the mean and standard deviation were calculated.

### Statistical analysis

Statistical analysis and graphical visualization of the data were performed using R (version 4.03) and GraphPad Prism 8.0 software. Flow data was analyzed using FlowJo software (10.8.1; FlowJo LLC). Correlations between variables use Pearson or Spearman coefficients. For all statistical calculations, a *p* value less than 0.05 was considered statistically significant.

## Result

### Pan-cancer analysis of PDHB expression

**Fig. 1:**
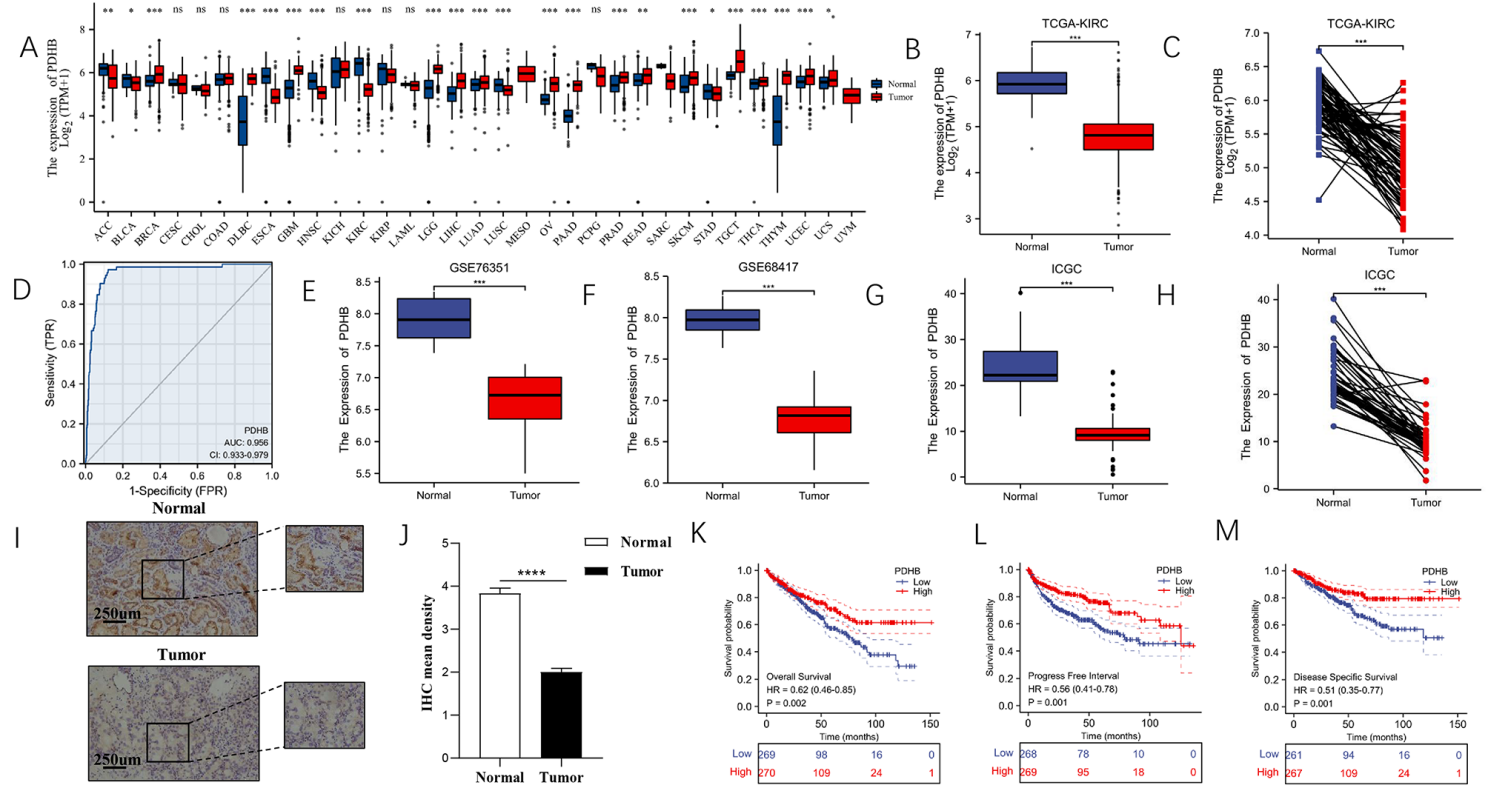
Differential expression and clinical prognosis of PDHB. **(A)** PDHB mRNA expression levels in TCGA tumor tissues and normal tissues. The mRNA expression **(B)** levels of PDHB in tumor and normal tissues in KIRC samples in the TCGA database, and mRNA **(C)** expression levels in tumor and paired normal tissues in KIRC samples in the TCGA database. **(D)** The diagnostic potential of PDHB for KIRC was estimated using theROC curve. Expression level of PDHB in GSE76351 **(E)** and GSE68417 **(F)**. The mRNA expression **(G)** levels of PDHB in tumor and normal tissue in KIRC samples from the ICGC database, and mRNA expression **(H)** levels in tumor and paired normal tissue in KIRC samples from the ICGC database. **(I-J)** Immunohistochemical staining of normal kidney tissue and KIRC tissue was performed. ​Kaplan-Meier curves demonstrating OS **(K)**, DFI **(L)** and DSS **(M)** for patients in the high and low PDHB expression groups of the KIRC sample. (* p < 0.05, ** p < 0.01, *** p < 0.001)

The overall research workflow is depicted in Figure S3. In this study, we investigated the role of the cuproptosis related gene PDHB in ccRCC. We first assessed the mRNA expression of PDHB in 33 human cancers and normal tissues using data from TCGA and GTEx datasets to investigate the potential involvement of PDHB in carcinogenesis and progression. Pan-cancer detection of PDHB expression showed that PDHB expression in tumor tissues was lower than that in normal tissues, including ACC, BLCA, ESCA, HNSC, LUSC and STAD. It was upregulated in another 20 tumors (Fig. 1A-B). In addition, we performed paired difference analysis to validate this finding from TCGA dataset using over 30 pairs of patient samples (Fig. 1C). The diagnostic potential of PDHB for ccRCC was estimated using the ROC curve to verify previous findings (Fig. 1D), it has high predictive accuracy for distinguishing tumor from normal tissue (AUC = 0.956). Moreover, we checked the mRNA expression of PDHB in GEO and ICGC datasets and found that GSE76351 and GSE6841 revealed decreased expression level of PDHB in tumor tissues (Fig. 1E-H). IHC was used to detect protein expression levels of PDHB in ccRCC tissue and normal kidney samples (Fig. 1I-J). The results showed that the expression level of PDHB was significantly lower in ccRCC tissues compared with adjacent normal tissues. Based on the difference of PDHB mRNA expression in cancer and paracancer samples, we found that PDHB was significantly correlated with better OS, DSS and PFS in ccRCC (Fig. 1K-M). In conclusion, our findings suggest that PDHB may be a favorable prognostic indicator for ccRCC.

### The correlation of PDHB and ccRCC mutations

**Fig. 2:**
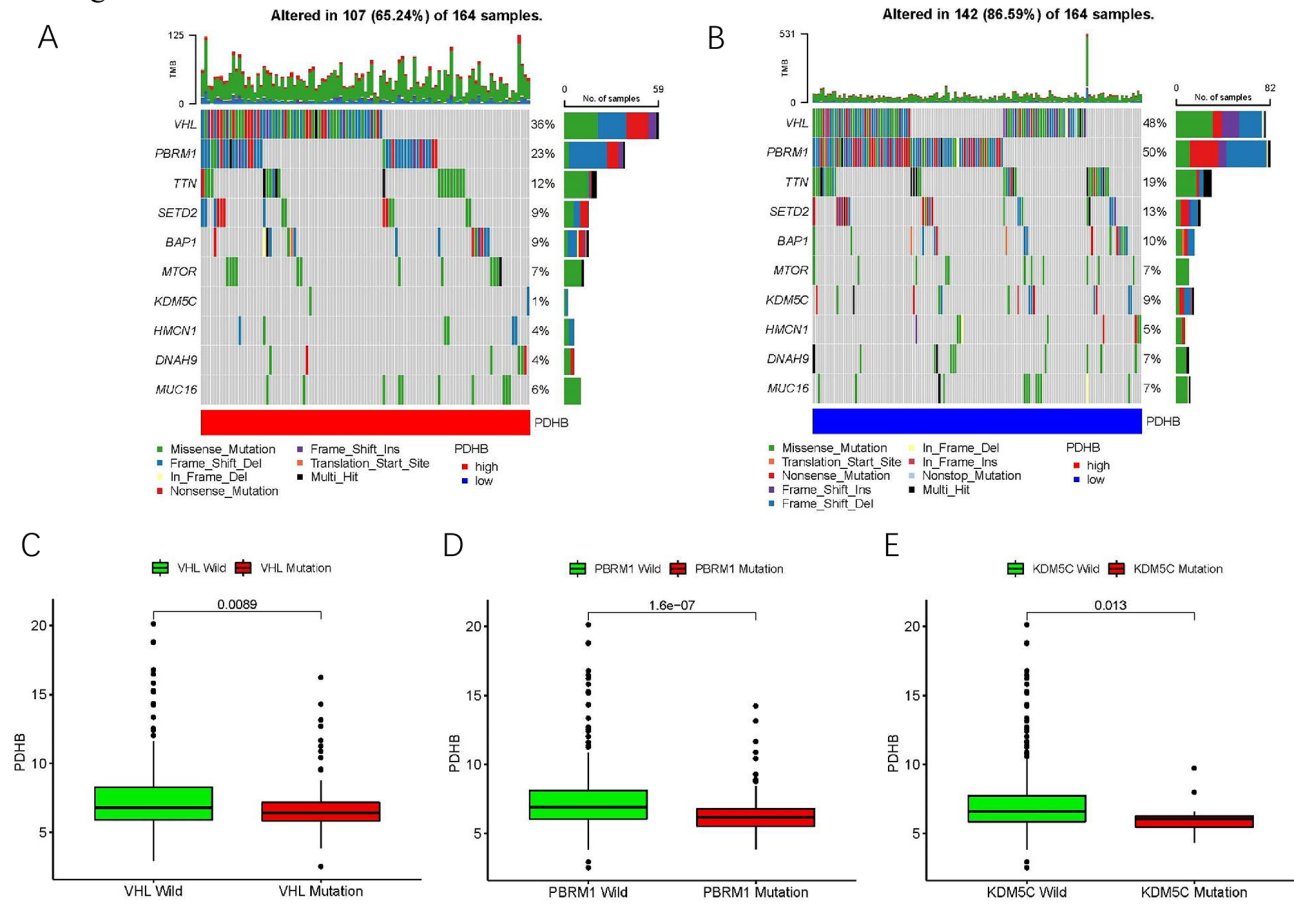
Molecular characterization of high- and low-PDHB expression subgroups. **(A-B)** Somatic mutation features of KIRC patients in high- and low-PDHB group. Differences in PDHB expression between **(C)** wild-type and VHL mutant subgroups, **(D)** PBRM1 mutant subgroups and **(E)** KDM5C mutant subgroups. (* p < 0.05, ** p < 0.01, *** p < 0.001)

To better understand the genetic changes, we identified the top 10 genes with the highest mutation rates in ccRCC samples subgrouped with higher and lower PDHB expression levels. Waterfall plots showed significant somatic mutations in both the higher and lower risk groups, with the highest mutation rate in VHL (Fig. 2A-B). In addition, we further investigated the difference in PDHB expression between wild-type and mutant-type. The results revealed that lower PDHB expression might lead to increased mutations in VHL (p = 0.0089), PBRM1 (p = 1.6e-07) and KDM5C (p = 0.013) (Fig. 2C-E).

### The relationship between PDHB and clinical features

**Fig. 3:**
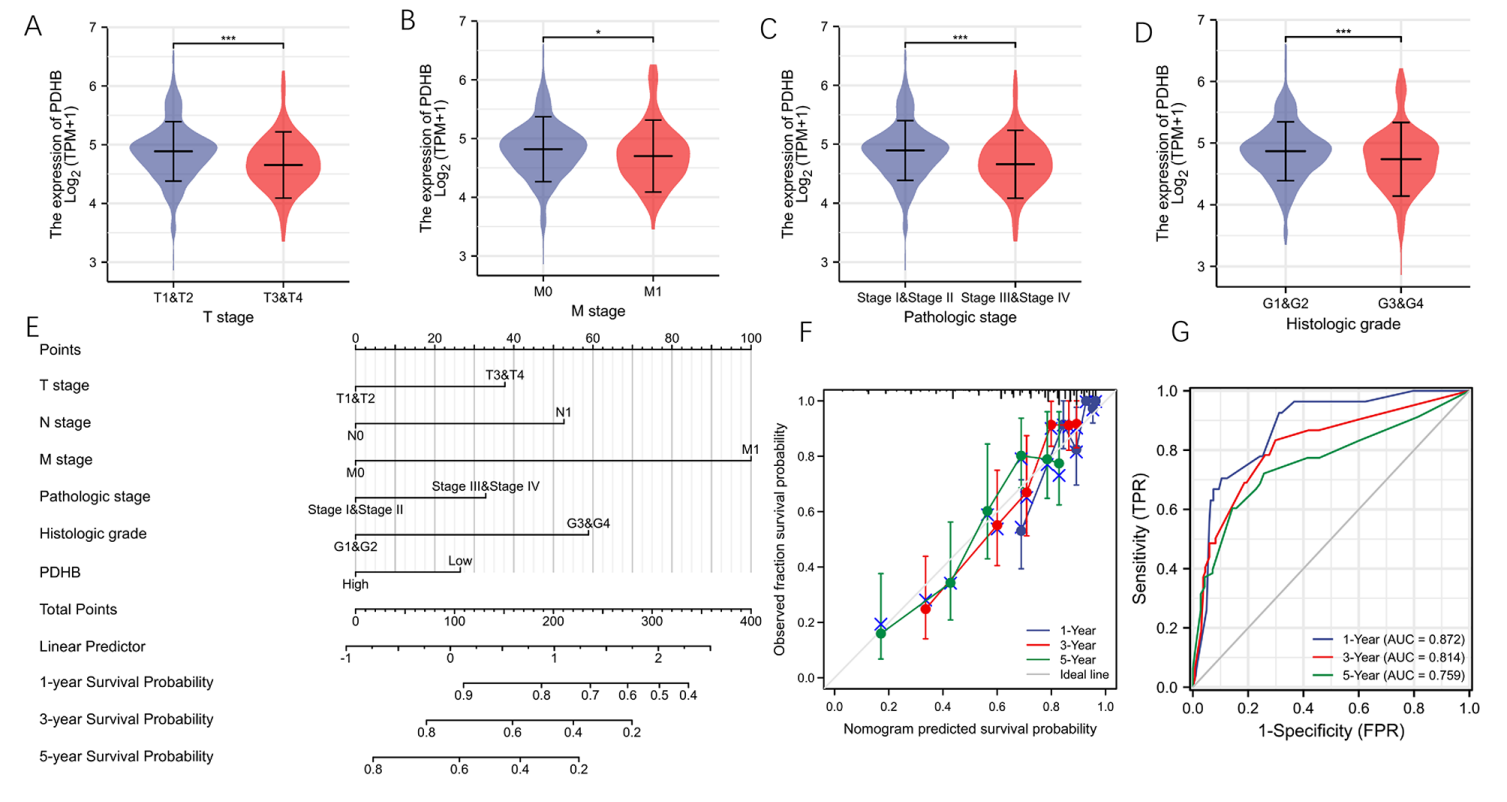
Correlation between the expression of PDHB and clinicopathological features of ccRcc. Correlation between expression and T-stage **(A)**, M-stage **(B)**, pathologic stage **(C)** and histologic grade **(D)** of ccRcc patients. **(E)** Nomogram for predicting the 1-, 3-, and 5-year overall survival for KIRC samples. **(F)** Calibration curves of the nomogram. **(G)** AUC curve of Nomogram for predicting the 1-, 3-, and 5-year overall survival

In mining the public TCGA dataset, we found that PDHB was significantly higher in T1 and T2 stages than that in T3 and T4 stages (p < 0.05) (Fig. 3A). The expression of PDHB in metastatic ccRCC was lower than that in non-metastatic renal clear cell carcinoma (p < 0.05) (Fig. 3B). In addition, the same results were concluded in histological stage and grade subanalysis (p < 0.05) (Fig. 3C-D). To further explore the prognostic potential of PDHB, a nomogram was created using a variety of clinical variables (Fig. 3E). The calibration curves showed that the predicted OS values at 1, 3 and 5 years were consistent with the actual data (Fig. 3F). The ROC curves were used to estimate the diagnostic potential of multiple factors for ccRCC, and AUCs of 0.872, 0.814 and 0.759 were observed (p < 0.05) (Fig. 3G).

### Co-expression networks of PDHB in ccRCC and gene set enrichment analyses

**Fig. 4:**
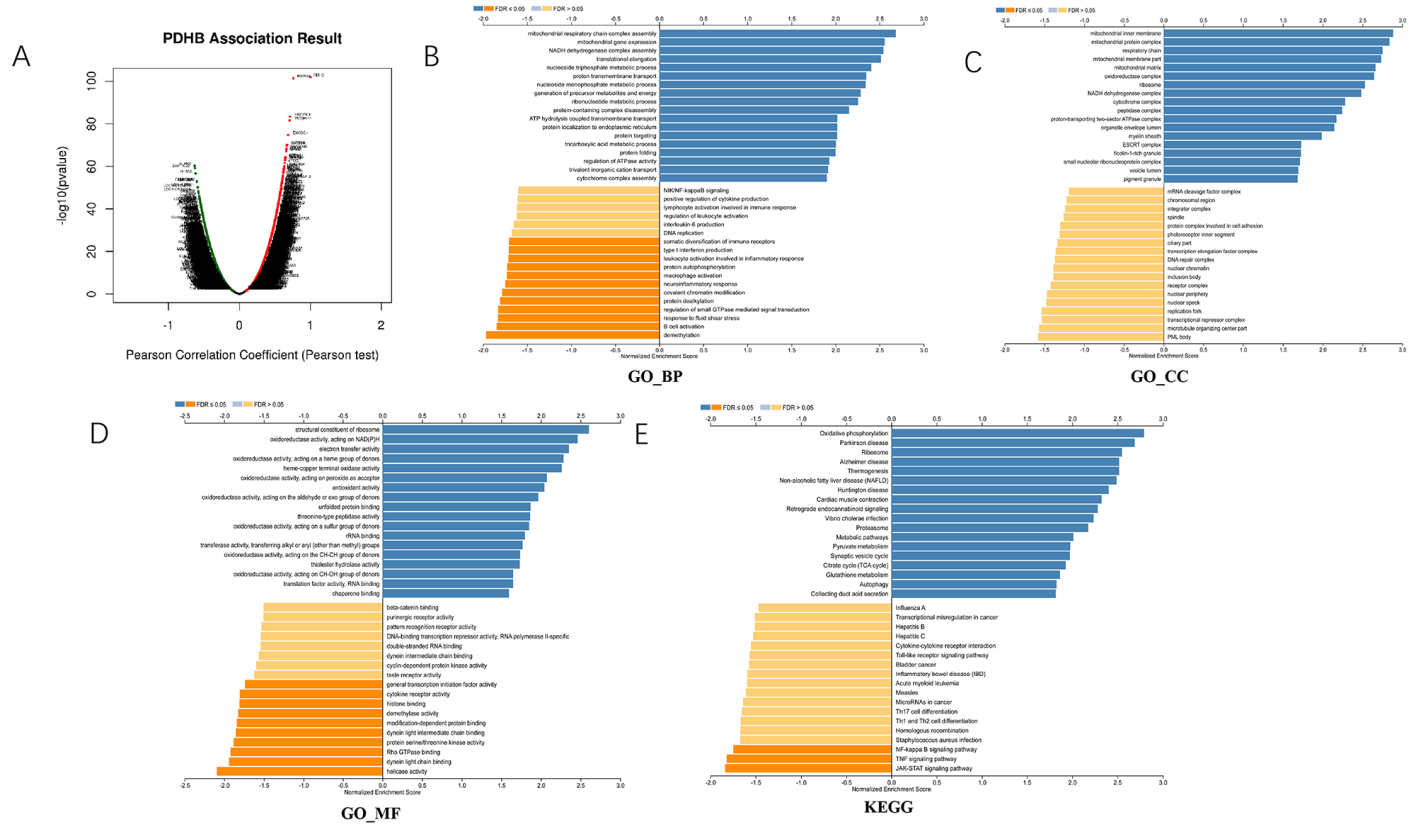
PDHB co-expression genes in KIRC (LinkedOmics). Figure 4. PDHB co-expression genes in KIRC (LinkedOmics). **(A)** The global PDHB highly correlated genes identified by the Pearson test in KIRC. **(B-D)** Significantly enriched GO: Biological process annotations and **(E)** KEGG pathways of PDHB in KIRC.

To better understand the biological significance of PDHBin ccRCC, we analyzed the co-expression network of PDHB in the TCGA-KIRC dataset and we used the “LinkFinder” module in LinkedOmics to explore the co-expression pattern of PDHB. As shown in Figs. 4A and 5526 genes (red dots) were positively correlated with PDHB and 7784 genes (green dots) were negatively correlated (p < 0.05). Heatmaps of the top 50 genes positively and negatively associated with PDHB are shown in Figure S1A and Figure S1B, respectively. According to Z-score data standardization, in the TCGA-KIRC database, PSMD6, UQCRC1, TMEM111, EXOSC7 and HIGD1A were positively correlated with PDHB. While ALPK2, SERTAD2, PHKA2, CA9 and FAM115C were negatively correlated with the expression of PDHB (the top five). Gene Ontology terminology annotation of GSEA showed that PDHB co-expressed genes were mainly involved in the mitochondrial respiratory chain complex assembly, mitochondrial gene expression, NADH dehydrogenase complex assembly and other functions (Fig. 4B-D). However, demethylation, B cell activation, response to fluid shear stress and regulation of small GTPase mediated signal transduction were inhibited. We also performed KEGG pathway analysis, which showed a high enrichment of co-expressed genes in the oxidative phosphorylation, Parkinson disease, ribosome and Alzheimer disease (Fig. 4E).

### Correlation of PDHB expression with immune cells and immune signatures

**Fig. 5:**
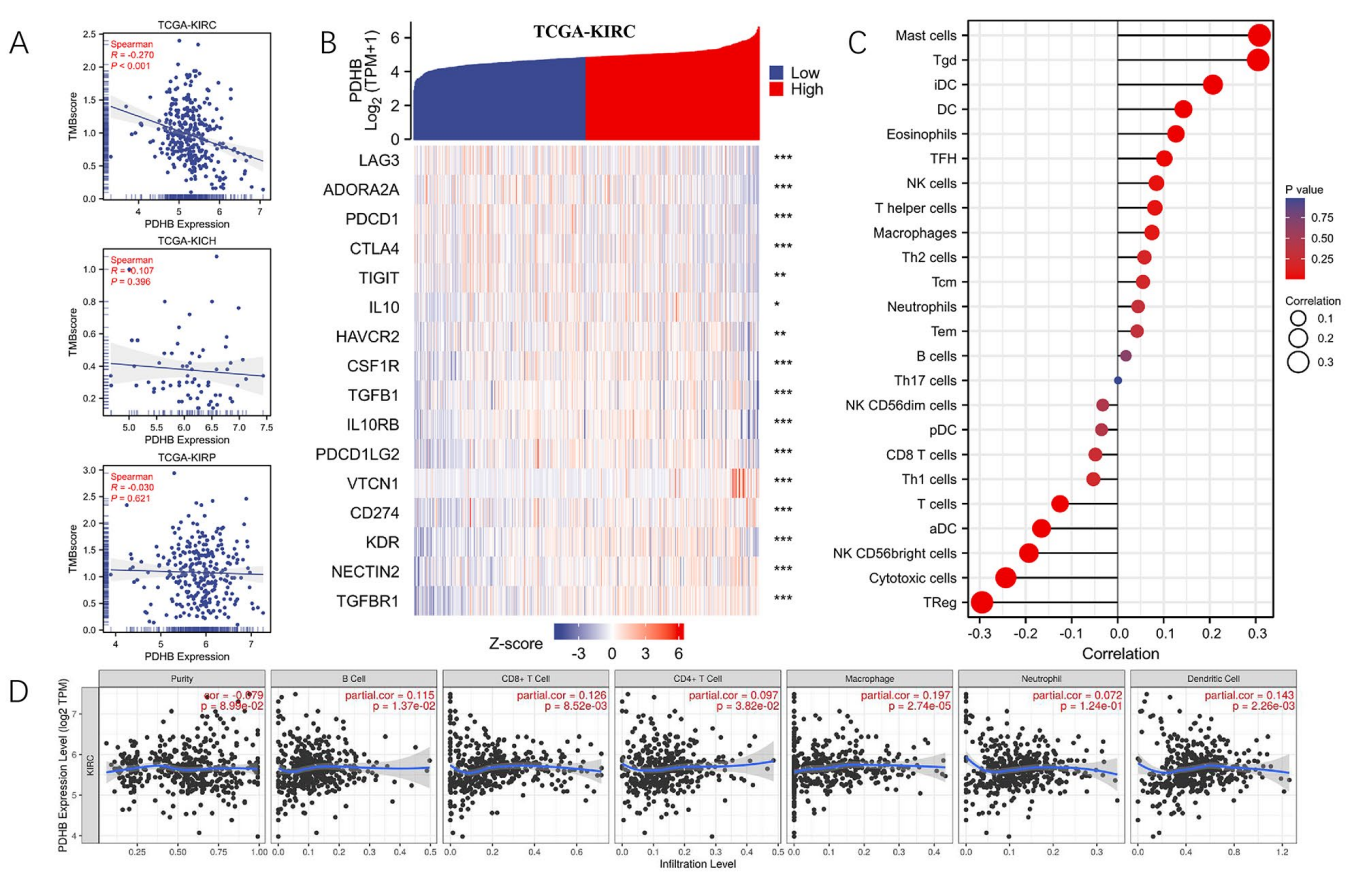
Correlation of PDHB expression with immune cells and immune signatures. **(A)** Correlation analysis of PDHB expression level with TMB score in TCGA-KIRC, TCGA-KICH and TCGA-KIRP. **(B)** Heatmap of immune checkpoint-associated genes are expressed in high- and low-PDHB subgroups. **(C)** The lollipop plot showed a correlation between PDHB expression and different tumor immune infiltrating cell ratios by ssGSEA (spearman test). **(D)** Correlation between PDHB expression and Six Types of immune infiltrating cells obtained by TIMER (Purity corrected Spearman test). (* p < 0.05, ** p < 0.01, *** p < 0.001)

It was unclear whether PDHB could affect the aggregation of immune cells in the tumor microenvironment to influence the prognosis of ccRCC. Figure 5 A showed the correlation between PDHB mRNA expression and TMB (tumor mutation burden) score of KIRC, KICH and KIRP. the result showed that PDHB was negatively correlated with KIRC. Furthermore, we evaluated the association of PDHB with immune checkpoints (LAG3, ADORA2A, PDCD1, CTLA4, TIGIT, IL10, HAVCR2, CSF1R, TGFB1, IL10RB, PDCDILG2, VTCN1, CD274, KDR, NECTIN2 AND TGFBR1). We found that the expression level of PDHB was significantly negatively correlated with LAG3, ADORA2A, PDCD1, CTLA4, TIGIT, IL10, HAVCR2, CSF1R, TGFB1, IL10RB, PDCDILG2 and KDR according to the TCGA-KIRC. (Fig. 5B; Table 1). Then, we investigated the correlation of PDHB and immune infiltration in KIRC by ssGSEA. Our results showed that the mRNA expression level of PDHB was significantly positively correlated with that of Mast cells, Tgd, iDC, DC and eosinophils. On the contrary, PDHB expression was negatively correlated with TReg, Cytotoxic cells, aDC and T cells (Fig. 5C; Table 2). Finally, we used the TIMER database to explore the correlation between PDHB and six major infiltrating immune cells in ccRCC (B cell, CD4 T cell, CD8 + T cell, neutrophil, macrophage and dendritic cell). The results showed that PDHB expression levels were positively correlated with the level of immune infiltration in B cells (r = 0.115, p = 1.37e-2), CD8 + T cells (r = 0.126, p = 8.52e-3), CD4 + T cells (r = 0.097, p = 3.82e-2), macrophage (r = 0.197, p = 2.74e-5), and dendritic cells (r = 0.143, p = 2.26e-3) (Fig. 5D). Tumor-infiltrating cells play a key role in tumor progression and affect the prognosis of patients with ccRCC. The results suggested that tumor immune escape and antitumor immunity might be involved in the oncogenic process of PDHB-mediated ccRCC.

**Table 1:** The correlation between the expressing of PDHB and immune checkpoint-associated genes. The p-values were calculated by using Pearson correlation analysis. Significant differences as p < 0.001

Variables	Correlation coefficient	P-value
ADORA2A	-0.213	**< 0.001**
CD274	0.297	**< 0.001**
CSF1R	0.087	**0.045**
CTLA4	-0.171	**< 0.001**
HAVCR2	0.047	0.28
IL10	0.082	0.059
IL10RB	0.051	0.234
KDR	0.279	**< 0.001**
LAG3	-0.193	**< 0.001**
PDCD1	-0.185	**< 0.001**
PDCD1LG2	0.178	**< 0.001**
NECTIN2	0.349	**< 0.001**
TGFB1	0.033	0.439
TGFBR1	0.452	**< 0.001**
TIGIT	-0.133	**0.002**
VTCN1	0.35	**< 0.001**

**Table 2:** PDHB and immune cell biomarkers in RCC were correlated by ssGSEA algorithm

Variables	Correlation coefficient	P-value
aDC	-0.166	**< 0.001**
B cells	0.018	0.678
CD8 T cells	-0.048	0.261
Cytotoxic cells	-0.243	**< 0.001**
DC	0.143	**< 0.001**
Eosinophils	0.127	0.003
iDC	0.207	**< 0.001**
Macrophages	0.074	0.084
Mast cells	0.308	**< 0.001**
Neutrophils	0.044	0.304
NK CD56bright cells	-0.193	**< 0.001**
NK CD56dim cells	-0.033	0.450
NK cells	0.084	0.051
pDC	-0.035	0.416
T cells	-0.125	**0.004**
T helper cells	0.081	0.061
Tcm	0.055	0.202
Tem	0.042	0.328
TFH	0.101	**0.019**
Tgd	0.305	**< 0.001**
Th1 cells	-0.053	0.220
Th17 cells	0.001	0.986
Th2 cells	0.058	0.178
TReg	-0.295	**< 0.001**
aDC	-0.166	**< 0.001**
B cells	0.018	0.678
CD8 T cells	-0.048	0.261
Cytotoxic cells	-0.243	**< 0.001**
DC	0.143	**< 0.001**
Eosinophils	0.127	**0.003**
iDC	0.207	**< 0.001**
Macrophages	0.074	0.084
Mast cells	0.308	**< 0.001**

p-values were calculated by using Pearson correlation analysis. Significant differences as p < 0.001

### The effect of copper on ccRCC

**Fig. 6:**
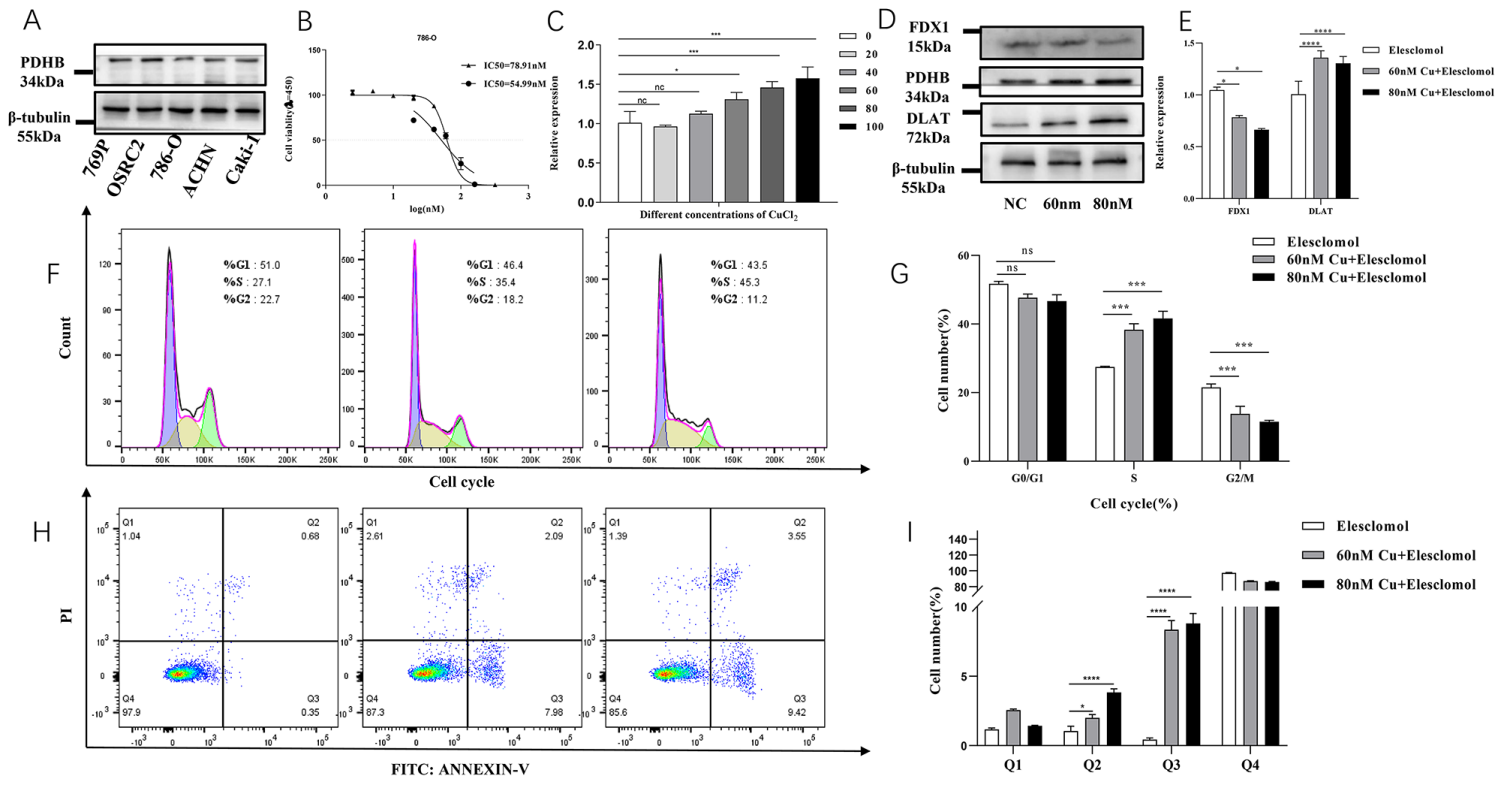
The effect of copper on KIRC. **(A)** Expression of PDHB in different renal carcinoma cell lines. **(B)** Cell viability of 786-O cells treated with copper chloride. **(C)** Expression of PDHB in different copper chloride concentrations. Expression levels of cuproptosis marker genes protein **(D)** (The membranes were trimmed reasonably before chemiluminescence (the original length is preserved. And the samples derive from the same experiment and that gels/blots were processed in parallel.) and mRNA **(E)** at three concentrations. Flow cytometric analysis of cell cycle **(F-G)** and cell apoptosis **(H-I)** in 786-O cells at three concentrations. (*p < 0.05, ** p < 0.01, *** p < 0.001)

We first examined the expression level of PDHB in different ccRCC cell lines. Western blotting results showed that PDHB expression level was the lowest in 786-O cells (Fig. 6A). Therefore, 786-0 cells were used for further experiments. After 48 h of exposure to copper chloride, the semi-inhibitory concentrations for the two concentration gradients were determined to be 78.91 nM and 54.99 nM, respectively (Fig. 6B). After treating 786-O cells for 48 h according to the second concentration gradient, total protein and total RNA were extracted. The expression level of PDHB was detected and gradually increased in 786-O cells treated with increasing doses of copper chloride (Fig. 6C), and the results were statistically significant above 60nM. We designed three groups (Elesclomol (NC), 60nM + Elesclomol, 80nM + Elesclomol) and conducted subsequent experiments after a 48-hour intervention period. After adding copper chloride, we tested the expression level of three key cuproptosis genes. Western blot showed increased level of PDHB and DLAT, but decreased level of FDX1 (Fig. 6D). Besides, PCR results also confirmed these findings .(Fig. 6E). Flow cytometry results showed that the growth of ccRCC cells was inhibited after adding copper chloride (Fig. 6F-I). Compared with the control group, the apoptosis of 786-O cells was significantly increased after copper chloride treatment (Fig. 6H-I). The cell cycle results showed that 786-O cells were blocked in S phase (Fig. 6F-G). In the previous study, we found that the ATR-FOXM1 pathway could inhibit cell proliferation through increased S-phase cells (Figure S2). Taken together, copper-treated ccRCC cells could promote cell death *via* cuproptosis.

### PDHB inhibited the proliferation of ccRCC cells

**Fig. 7:**
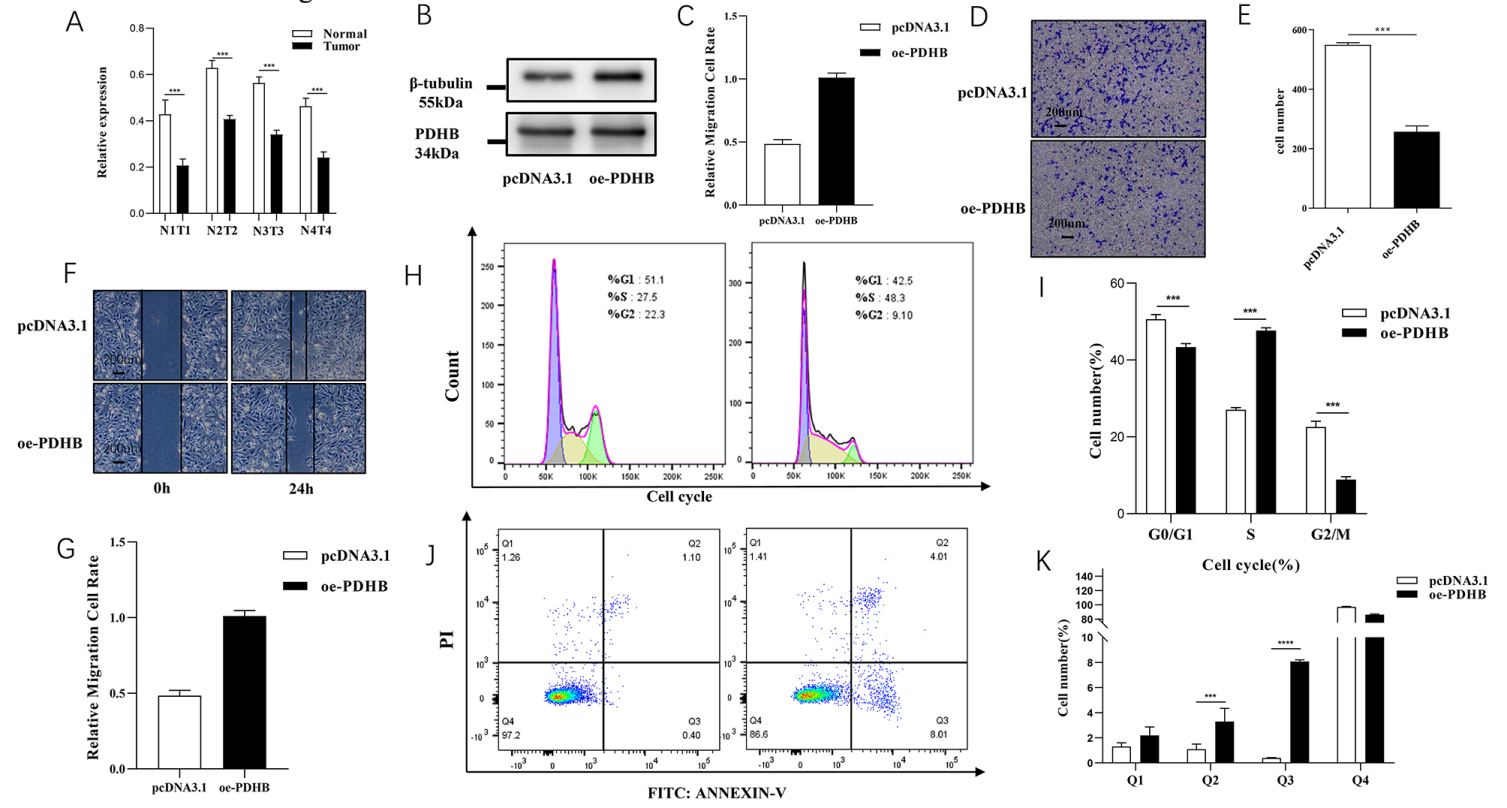
PDHB inhibited the proliferation of renal clear cell carcinoma on 786-O cells. **(A)** PDHB downregulated in ccRCC tissues at the mRNA level. Western blot **(B)** (The membranes were trimmed reasonably before chemiluminescence (the original length is preserved).) and RT-PCR **(C)** were used to detect the transfection efficiency of ccRCC cells transfected by PDHB overexpression plasmid. Cell migration after overexpression of PDHB was evaluated by transwell assays **(D-E)** and wound healing **(F-G)**. The proliferation level after overexpression of PDHB was evaluated by flow cytometry (Cell Cycle **(H-I)**, Cell apoptosis **(J-K)**). (*p < 0.05, ** p < 0.01, *** p < 0.001)

The qRT-PCR results showed that lower expression of PDHB in ccRCC tissues compared with normal tissues (Fig. 7A). To investigate the role of PDHB in ccRCC, we made an overexpression model of PDHB in the 786-O cell line. We used qRT-PCR and Western blot to detect the expression levels of PDHB (Fig. 7B-C). Next, the effect of PDHB expression on ccRCC cell migration and invasion was investigated *via* transwell (Fig. 7D-E) and wound healing migration experiments (Fig. 7F-G). After overexpression of PDHB, the cell migration and invasion capacity of 786-O cells was significantly reduced. To investigate whether PDHB inhibits cell proliferation by influencing the cell cycle, flow cytometry experiments were conducted. We found that overexpression of PDHB resulted in a significant increase in the number of S-phase cells, as well as a significant increase in the number of late apoptotic(Q2) and early apoptotic(Q3) cells (Fig. 7H-K). Flow cytometry results indicate that after overexpressing PDHB, the majority of cells are inhibited in the S phase of the cell cycle. The results indicate that overexpressing PDHB can inhibit cell proliferation.

## Discussion

RCC is one of the common malignant tumors of the urinary system, accounting for more than 80% of kidney malignancies and 2-3% of adult malignancies [2, 33]. Most ccRCC patients are diagnosed at early-stage, but almost 20–30% ccRCC patients are diagnosed metastatic lesions. In addition, 20–50% of early-stage ccRCC will progress to metastatic diseases [34]. The main treatment modality for early-stage ccRCC is surgery (partial nephrectomy and radical nephrectomy). Approximately 10–20% of early-stage ccRCC would recur or metastasize after surgery [35–37]. However, advanced ccRCC indicates a poor survival rate. In recent years, the treatment of advanced ccrCC has shifted from cytokine therapy [38, 39] to targeted drug therapy. Targeted therapeutic agents, including vascular endothelial growth factor, VEGF inhibitors and mammalian target of rapamycin mTOR [40–43]. With the development of various new drugs, immune checkpoint inhibitors have brought a new light to patients with advanced ccRCC [44]. For example, PD-L1/PD-L2 inhibitors Nivoluumab [45], Ipilimumab [46] and so on. However, the OS and PFS of advanced ccRCC is still poor, new treatments are needed for advanced ccRCC patients.

Recent studies have identified a novel form of cell death caused by copper called copper-induced cell death (cuproptosis) [15]. The mechanism of cuproptosis exhibits a profound interrelation with mitochondrial respiration. Surplus intracellular copper is capable of transportation to the mitochondria through ion carriers,where it forms direct bonds with the lipid acylated components of the tricarboxylic acid cycle. This interaction subsequently triggers the aggregation of esterified proteins and the disruption of iron-sulfur cluster proteins, thereby initiating a state of proteotoxic stress that ultimately culminates in cellular demise [16]. But a previous study found that copper increased in the serum of cancer patients and was involved in tumor cell proliferation and angiogenesis. Juloski’s research has shown that copper builds up in areas of colon cancer tumors [47]. Saleh et al. found that copper was significantly increased in the serum of prostate cancer [48] patients. Kucharzewski et al. discovered that copper accumulates in the nuclear of breast cancer cells [49]. It has also been reported that increased serum copper in cancer patients is significantly associated with cancer grade and chemotherapy resistance [50]. So far, the metabolism of copper in ccRCC remains unclear.

In this study, we performed a pan-cancer analysis of PDHB using the TCGA database. And we found that PDHB was lower expressed in a variety of cancers. Furthermore, our investigations revealed a significant correlation between diminished PDHB expression and unfavorable prognostic indicators such as OS, DFI, and PFI, as evident from our survival analysis specifically conducted on TCGA-KIRC data. To investigate the role of PDHB in ccRCC progression, we explored the intricate interplay between clinicopathological parameters encompassing the stage and grade of ccRCC, and the expression levels of PDHB. Specifically, PDHB exhibited a marked decrease in cases of advanced malignancies, suggesting a potential role in the progression of ccRCC. PDHB showed a good predictive effect for ccRCC according to the ROC curve. To get a comprehensive understanding of the function of PDHB, we performed enrichment analysis of co-expressed genes (p < 0.05) of PDHB. The results showed that the expression of PDHB was associated with mitochondrial respiratory chain complex assembly, mitochondrial gene expression and NADH dehydrogenase complex assembly. KEGG pathway enrichment analysis revealed that PDHB expression may be associated with multiple pathways, including oxidative phosphorylation, Parkinson disease, ribosome and Alzheimer disease, among others. In the analysis of PDHB molecular characterization, we found that lower expression of PDHB may lead to increased VHL, PBRM1 and KDM5C mutations. We also investigated the effect of PDHB on the immune microenvironment of ccRCC. We found that PDHB was significantly associated with ccRCC immune cells and immune checkpoints. Knockdown of lipid acylated protein targets can save cells from copper-induced death [15]. PDHB as an isoform of the pyruvate complex is a gene in mitochondria that encodes a lipid acylated protein [23]. Our cell experiments also showed that both copper and PDHB inhibited the proliferation and migration of ccRCC cells.

Numerous previous studies have identified the importance of cuproptosis-related genes in the ccRCC immune microenvironment and can predict OS in patients with ccRCC [51, 52]. Chen et al. and Zhang et al. reported that higher expression of FDX1 was associated with a better prognosis of renal clear cell carcinoma and could be used as a potential prognostic indicator and therapeutic target for ccRCC [53, 54]. So far, the effect of PDHB expression on renal clear cell carcinoma remains unclear.

While our study has provided valuable insights, it’s important to note its limitations. These limitations relate to our data sources and our understanding of mechanisms. Firstly, much of our data comes from databases like TCGA and GEO. These databases offer abundant information, they come with certain issues. Differences in experimental methods, sample preparations, and technology platforms can introduce some heterogeneity and bias into our analysis. Secondly, while we suggest that PDHB might be a biomarker for ccRCC, this is based on existing data. To fully confirm this finding, we need larger datasets and comprehensive clinical prognosis information. Without such datasets, we can’t fully establish PDHB’s predictive ability. Thirdly, we investigated PDHB’s role in cell cycle regulation, but our understanding is still somewhat limited. Even though we observed an upregulation of the ATR gene after copper treatment, the exact details of how PDHB and cell cycle regulation interact require further experimentation and research to clarify. Fourthly, our study suggests a potential connection between PDHB and immune regulation, but we may not have fully grasped the complexity of immune interactions in ccRCC. To comprehensively understand PDHB’s impact on immunity, more diverse types of clinical samples are needed for analysis. In summary, while our study has shed light on PDHB’s role in ccRCC, it’s crucial to acknowledge these limitations. Addressing these limitations by utilizing more diverse data sources and conducting deeper research will enhance the credibility and clinical relevance of our findings.

## Conclusion

Our findings suggest that decreased expression of PDHB is closely associated with an elevated risk of tumor progression in ccRCC. Furthermore, our study indicates a potential involvement of PDHB in the cuproptosis process within ccRCC, thereby positioning it as a promising prognostic predictor for this malignancy. Leveraging the insights gained from this study, targeting PDHB at the molecular level emerges as a promising avenue for developing innovative therapeutic strategies tailored for patients with advanced ccRCC.

### Electronic supplementary material

Below is the link to the electronic supplementary material.

Supplementary Material 1

Supplementary Material 2

Supplementary Material 3

Supplementary Material 4

## Data Availability

The datasets used in the current study are openly available in TCGA (http://cancergenome.nih.gov/, accessed on 12 July 2022) database, UCSC Xena data portal (https://xenabrowser.net/, accessed on 15 July 2022), LinkedOmics (http://www.linkedomics.org) and The Tumor Immune Estimation Resource (TIMER, https://cistrome.shinyapps.io/timer/). Two microarray datasets GSE76351 and GSE68417 were downloaded from GEO (https://www.ncbi.nlm.nih.gov/geo/, accessed on 1 July 2022) database. All data and materials are available from the corresponding authors upon request.

## Abbreviations

ACC: Adrenocortical carcinoma
BLCA: Bladder Urothelial Carcinoma
BP: Biological Process
CC: Cellular Component
ccRCC: Clear Cell Renal Cell Carcinoma
DSS: Disease Specific Survival
ESCA: Esophageal Carcinoma
FDR: false discovery rate
GEO: Gene Expression Omnibus
GEPIA: Gene Expression Profiling Interactive Analysis
GO: Gene Ontology
GSEA: Gene Set Enrichment Analysis
GTEx: Genotype-Tissue Expression
HNSC: Head and Neck squamous cell carcinoma
KEGG: Kyoto Encyclopedia of Genes and Genomes
KICH: Kidney Chromophobe
KIRC: Kidney renal clear cell carcinoma
KIRP: Kidney renal papillary cell carcinoma
LUSC: Lung squamous cell carcinoma
MF: Molecular Function
OS: Overall survival
PFI: Progress Free interval
ROC: Receiver Operating characteristic
ssGSEA: single sample gene set enrichment analysis
STAD: Stomach adenocarcinoma
TCGA: The Cancer Genome Atlas
TIMER: Tumor Immune Estimation Resource
