# Supplementary figures and images for: Cuproptosis related gene PDHB is identified as a biomarker inversely associated with the progression of clear cell renal cell carcinoma
Source: BMC Cancer. 2023 Aug 28;23:804. doi: 10.1186/s12885-023-11324-0 (PMC10464351; doi:10.1186/s12885-023-11324-0)

Figure 6A

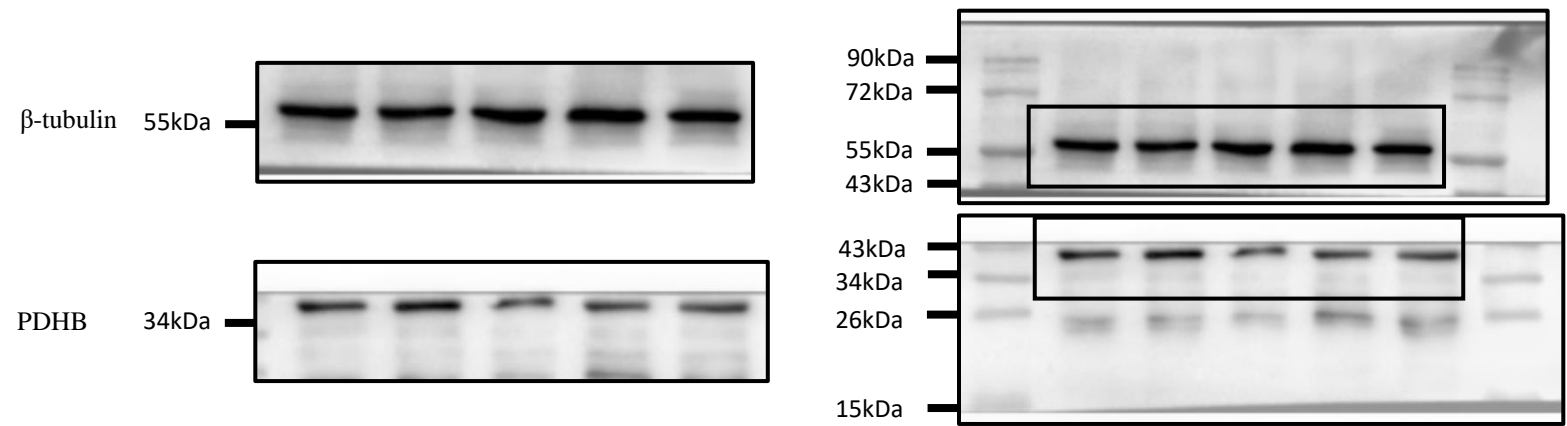

Figure 6D

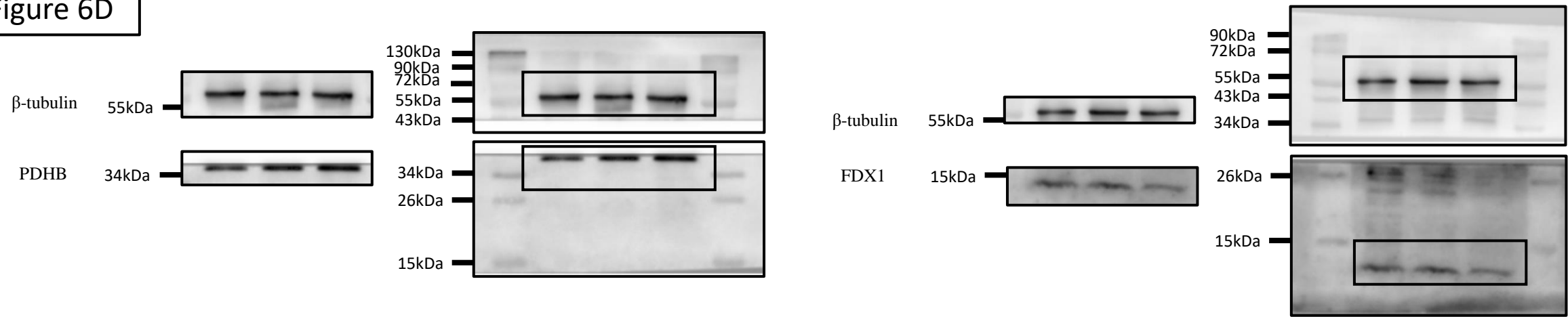

Figure 6D

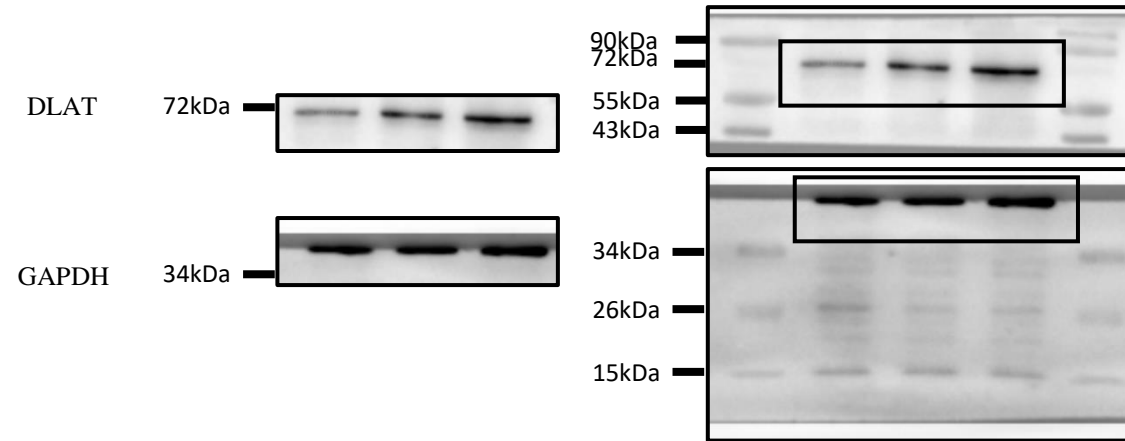

Figure 7B

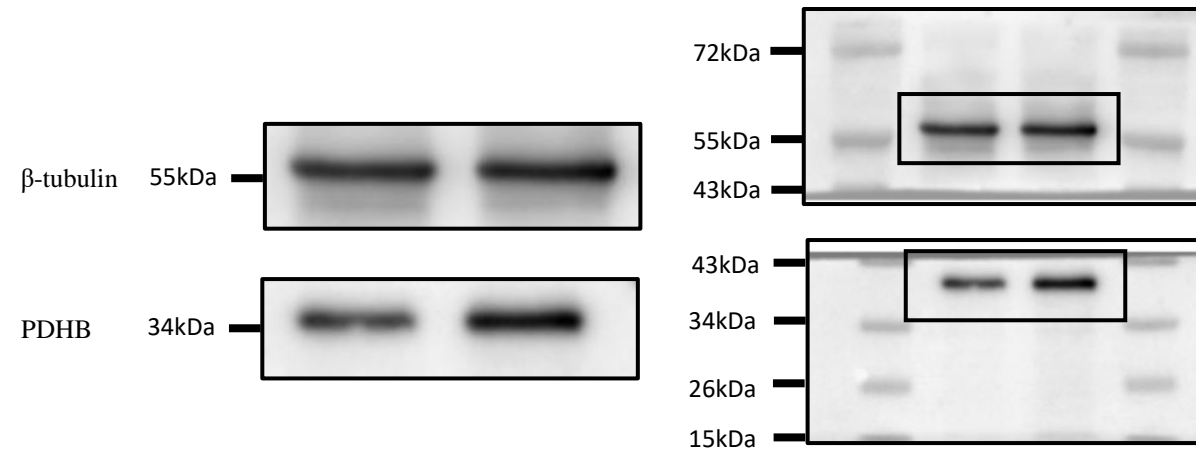

Supplement: Supplementary file 1 — Supplementary Material 1 [file 12885_2023_11324_MOESM1_ESM.pdf]

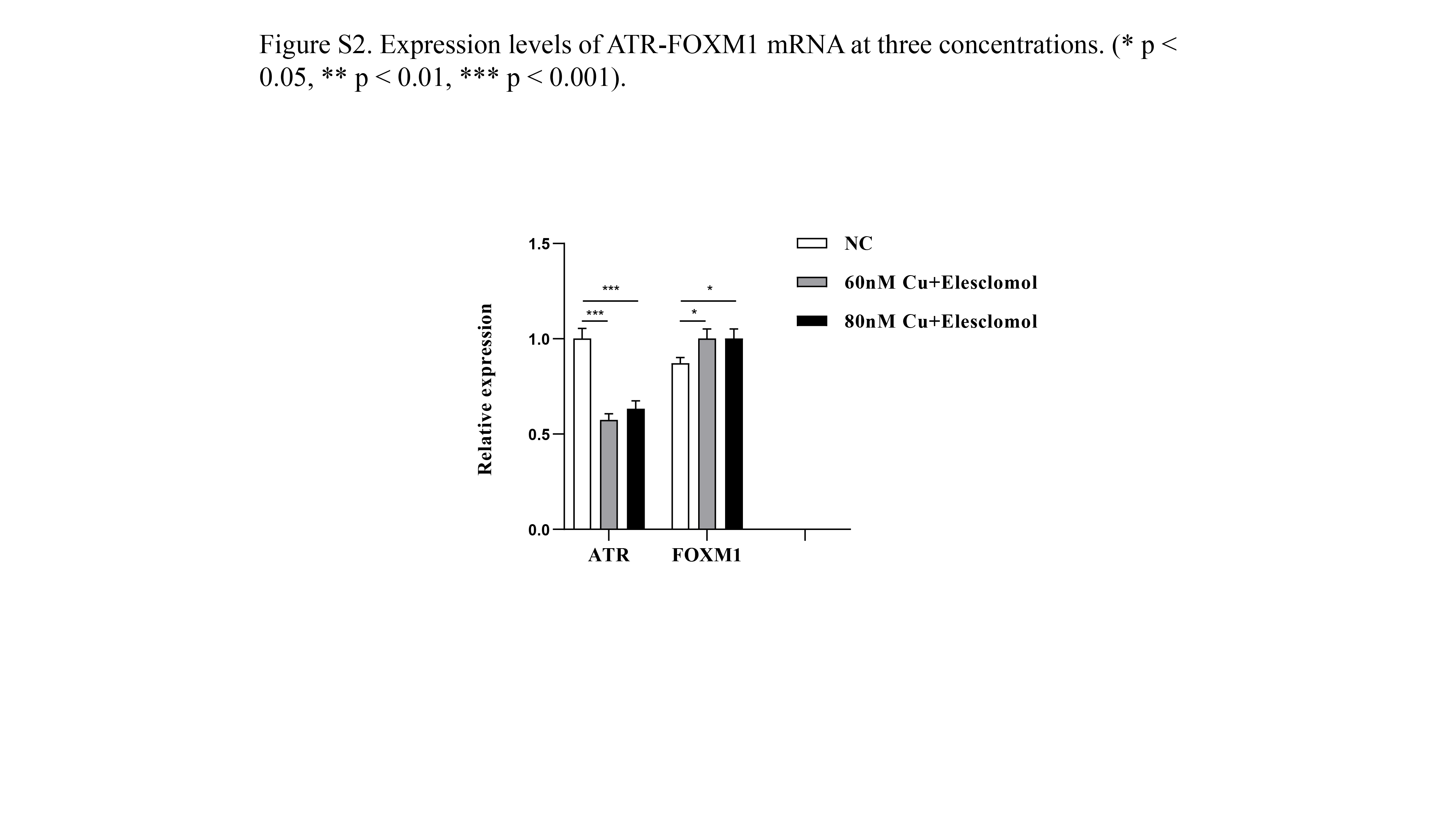

Supplement: Supplementary file 2 — Supplementary Material 2 [file 12885_2023_11324_MOESM2_ESM.png]

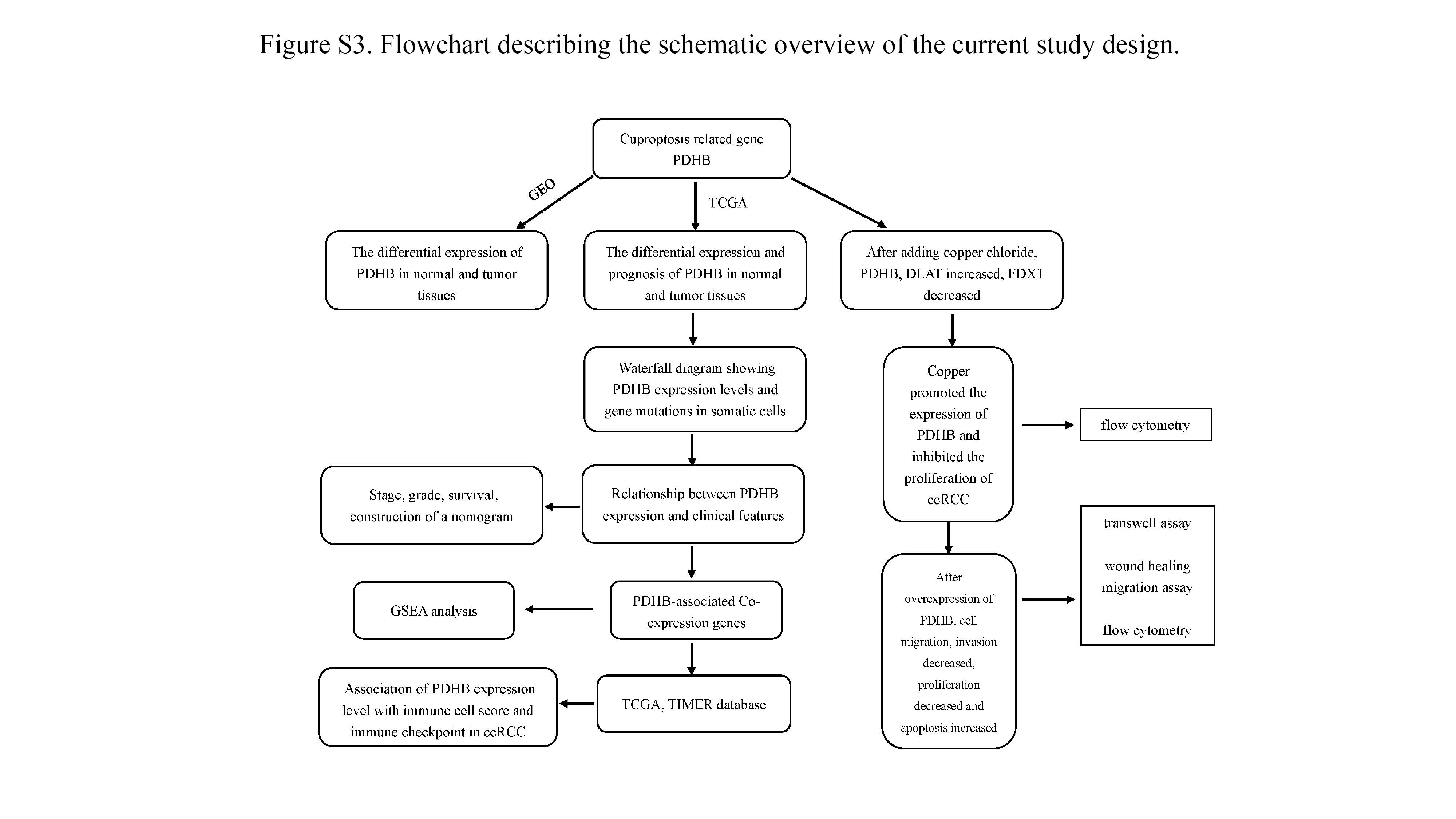

Supplement: Supplementary file 3 — Supplementary Material 3 [file 12885_2023_11324_MOESM3_ESM.png]

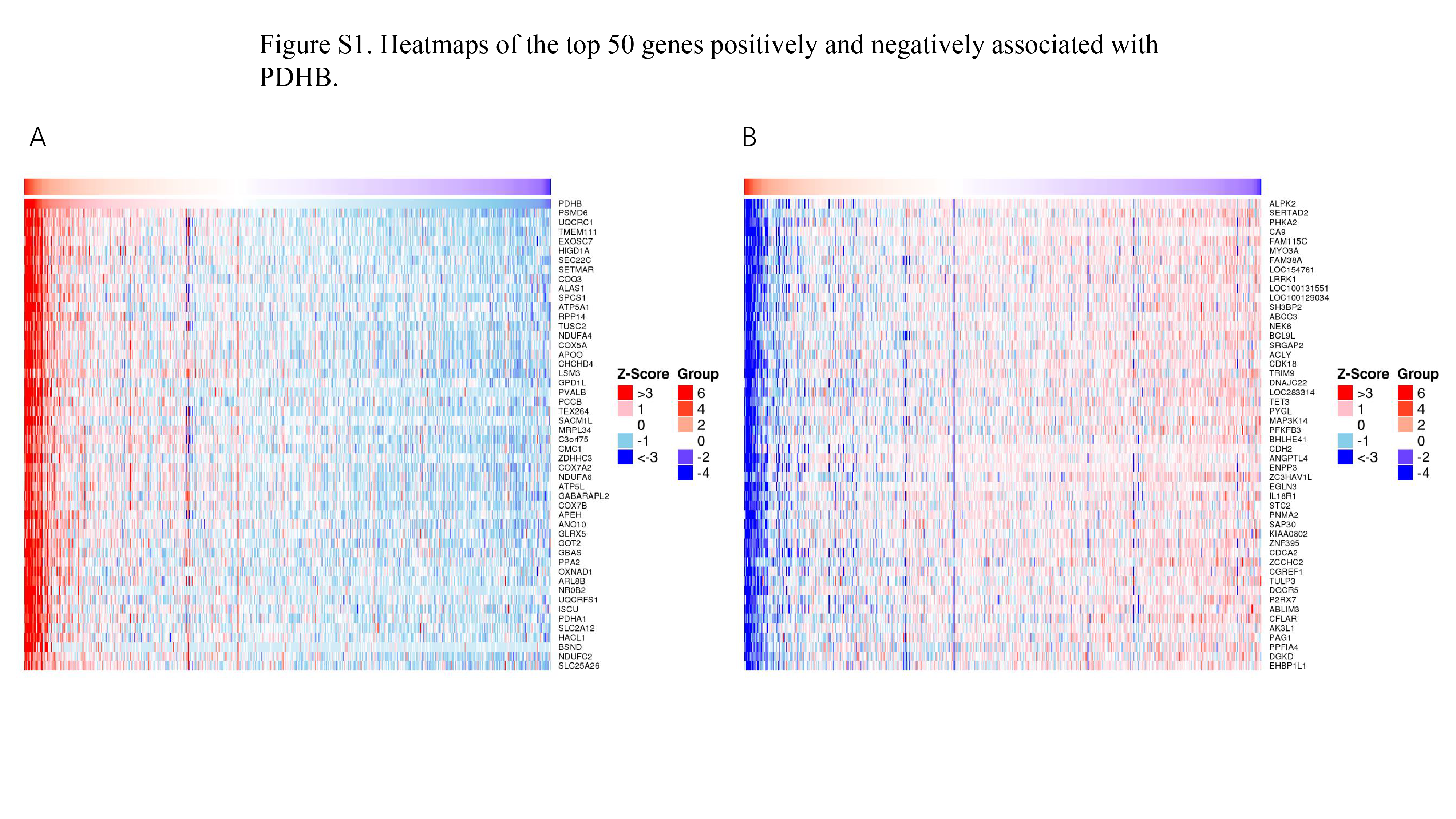

Supplement: Supplementary file 4 — Supplementary Material 4 [file 12885_2023_11324_MOESM4_ESM.png]
